# From oxygen shortage to neurocognitive challenges: behavioral patterns and imaging insights

**DOI:** 10.3389/fcogn.2024.1468306

**Published:** 2024-11-05

**Authors:** Alberto Zani, Yldjana Dishi, Alice Mado Proverbio

**Affiliations:** ^1^School of Psychology, Vita-Salute San Raffaele University, Milan, Italy; ^2^Department of Psychology, University of Milan Bicocca, Milan, Italy

**Keywords:** normobaric and hypobaric hypoxia, high altitude, working memory, attention, alertness, brain and behavior, EEG/ERPs, neuroimaging

## Abstract

Environmental hypoxia, resulting from reduced oxygen supply, poses a significant risk of dysfunctioning and damaging the neurocognitive system, particularly in relation to anxiety and stress. Inadequate oxygenation can lead to acute and chronic brain damage. Scholars used behavioral, hemodynamic, and electromagnetic neurofunctional techniques to investigate the effects of normobaric and hypobaric hypoxia on neurocognitive systems. They found a correlation between hypoxia, altered psychomotor responses, and changes in EEG alpha, theta, beta, and gamma rhythms, which affect spatial attention and memory. Hypoxia affects event related potential (ERP) components differently depending on latency. Perceptual responses N1 and P2 remain largely unaffected, while the amplitudes of preattentive MMN, vMMN, and P3a are significantly altered. Late latency components related to attention, particularly P3b, are also altered. These changes illustrate the spectrum from sensory detection to more complex cognitive processing, highlighting the brain's efficiency in managing information. Interestingly, the amplitudes of P3b, ADAN and CNV can increase with increased cognitive demands in hypoxia. This suggests a compensatory response. Prolonged exposure exacerbates these effects, resulting in compensatory delayed behavioral responses and alterations in behavioral monitoring and conflict inhibitory control, as reflected by reduced amplitudes in some attention related ERP components, including N2, N2pc, and ERN. Thus, neurocognitive function and integrity are under stress. ERP sources and hemodynamic images reveal that vulnerable brain regions include the frontal prefrontal cortices, hippocampus, basal ganglia, and parietal and visual cortices, which are essential for attention related processes like decision making and spatial memory. The auditory system appears less affected.

## Introduction

The process of breathing involves bringing in oxygen, which is necessary for aerobic metabolism, which takes place in the cell's mitochondria. When oxygen is inadequate, the organism is in a state of so-called hypoxia. The latter is distinct from anoxia, which occurs when the oxygen supply to our body or brain is totally cut off. Up to the troposphere, which is at a height of around 12,000 m (39,370 feet: ft), the air's oxygen content stays constant at 20.9%, although pressure drops as one ascends higher. While hypoxemia brought on by anoxia is concerned with the decline of oxygen saturation in arterial blood that changes with the proportion of oxygen breathed, environmental hypoxia is defined as the drop of *alveolar oxygen pressure* (PaO_2_) and arterial partial *pressure of oxygen* (PO_2_) (Virués-Ortega et al., 2004). Like any survival barrier, hypoxia must be compensated for, and this is done by increasing breathing or triggering a *high ventilation response* (HVR). This response is indicated as VE40, denoting a 40 mm Hg air exhalation volume under alveolar normoxia.

Prolonged exposure to high altitude (HA) leads to a drop in HVR and produces a process of acclimatization, i.e., a form of adaptation: in reality, for societies living at high elevations, lower HVR and reduced susceptibility to hypoxia have been established. The body is severely affected by the spectrum of disorders resulting from this condition, both in terms of psychomotor performance and physiological functions. The 19th century saw the emergence of problems associated with hypoxia and/or anoxia (Coksevim et al., 2007; Grocott et al., 2009). These issues were brought about by mountain climbers who reached high mountain summits exceeding 2,000 m [about (~) 6,652 ft] above sea level (a. s. l.). Hypoxia can be tissue-based, meaning it affects a particular group of cells and consequently a whole body, or generalized, meaning it affects the entire body and results in low blood oxygen levels.

Additional literature offered evidence that exposure to altitude decreases one's absolute thresholds for touch, smell, light vision, taste, and CO_2_ detection as well as one's discrimination capacity. The decreases range from 25 to 40% and are attributed to hypoxia because the effects return rapidly with oxygen administration (Virués-Ortega et al., 2004). Anoxia is also the cause of hallucinatory experiences, which are perceptual changes. In 1933, mountaineers who climbed Everest reported visual hallucinations, including seeing a nonexistent companion, “feeling” accompanied, or a case of red snowflakes above 6,900 m (~22,638 ft). The decreased thresholds for differentiating between scents, light, and painful stimuli could potentially provide an explanation for the hallucinations experienced by climbers without additional oxygen above 6,000 m (19,685 ft).

The brain, relative to its size and metabolic activity, exhibits a pronounced sensitivity to reductions in oxygen supply, utilizing ~20% of the body's total oxygen intake. Consequently, hypoxia is a critical factor in this context, with significant implications for psychopathological and neuropathological outcomes, as well as its detrimental effects on both fundamental and complex cognitive functions (Bonkowsky and Jong-Hyun, 2018).

Our review is centered on the neurocognitive effects of hypoxia, or reduced oxygen availability, in various environmental conditions, such as e.g. decompression of aircraft cockpits, diving, respiratory insufficiency, etc., but more specifically in high-altitude mountain environments. It categorizes hypoxia into two types: normobaric, where oxygen levels are low but pressure remains constant, and hypobaric, characterized by low pressure at altitude. It emphasizes that both forms have distinct but related effects on the full range of neurocognitive functions (sensory, perceptual, vigilance, expectancy, problem solving, deductive reasoning, decision making and others). To disentangle possible differences between the effects of acute exposure and long-term or chronic exposure to hypoxia, these effects are discussed separately. Acute mountain sickness (AMS) is considered and it is noted that acclimatization, physical fitness and respiratory regimes can improve cognitive function in altinauts. In addition, behavioral and neuroimaging markers such as EEG, ERPs and fMRI are reviewed to assess cognitive changes. We will critically analyze the brain regions affected, including the prefrontal cortex and hippocampus. We will highlight the need to understand the risks of hypoxia on cognitive performance and brain health.

### Relationships of SaO_2_ and SpO_2_ with hypobaric and normobaric hypoxia

As anticipated, let us briefly discuss the nature of both SaO_2_ (arterial oxygen saturation) and SpO_2_ (peripheral capillary oxygen saturation) and the differences between the two types of hypobaric and normobaric hypoxia that occur physiologically.

SaO_2_ and SpO_2_ are important indicators in experimental studies for several reasons.

Both SaO_2_ and SpO_2_ provide information about the oxygen-carrying capacity of blood. SaO2 refers to the percentage of hemoglobin saturated with oxygen in arterial blood, and SpO_2_ refers to the same measurement estimated non-invasively using a pulse oximeter clipped onto a fingertip or earlobe. Both indicators reflect how effectively oxygen is being transported to tissues by red blood cells.

Hypoxia refers to a deficiency in the amount of oxygen reaching tissues. By measuring SaO_2_ and SpO_2_ levels, it can be assessed whether an individual has adequate oxygenation. Normal levels are typically above 95%. Levels below this threshold may indicate varying degrees of hypoxemia and the need for medical evaluation for administration of supplemental oxygen, mechanical ventilation, or other interventions aimed at correcting hypoxia. In summary, SaO_2_ and SpO_2_ are essential for evaluating oxygenation and identifying hypoxia, critical for individual safety and effective treatment in various physiological and clinical scenarios.

*Normobaric hypoxia* (NH) occurs when the fraction of inspired oxygen (FiO_2_) is reduced while the total barometric pressure remains stable. This means that the percentage of oxygen in the air we breathe is lower, which directly affects the amount of oxygen available for the body. An example of this would be being in a room where the oxygen concentration is artificially decreased. NH may be induced by lowering the partial pressure of oxygen (PiO_2_) in the ambient air (Conkin and Wessel, 2008).

*Hypobaric hypoxia* (HH), on the other hand, occurs when the barometric pressure itself is lowered, while the FiO_2_ remains unchanged. This can happen at high altitudes where there is less air pressure overall, resulting in a lower availability of oxygen despite the proportion of oxygen in the air being the same as at sea level.

According to some interesting findings by Tsarouchas et al. (2008), in situations of HH (such as at the indicated high altitudes where the atmospheric pressure is lower), the blood oxygen saturation often falls below 75%. This means that the ability of hemoglobin to carry oxygen is significantly impaired in such environments, reflecting the challenges of obtaining an adequate supply of oxygen under these conditions.

Both types of hypoxias can be simulated or studied by altering the ambient conditions. For NH, the oxygen fraction in the air (FiO_2_) can be decreased, effectively lowering the PiO_2_, while for HH lower barometric pressures can be created, such as in altitude chambers. Therefore, both approaches are commonly used in research to examine the physiological effects of reduced oxygen availability on the body. This highlights the various methods by which the impacts of hypoxia in controlled environments can be investigated.

The two physical sources of hypoxia mentioned above are thought to depend on PiO_2_ for physiological responses to hypoxic stimuli, independent of changes in FiO_2_ or barometric pressure.

In this regard, a systematic meta-regression analysis of the effects of acute hypoxia on performance across a wide range of neurocognitive functions found that there were no significant differences between normoxic and hypoxic conditions, regardless of whether the hypoxic exposure occurred in normobaric or hypobaric mode. This conclusion was shown to be dependent on the low level of PaO_2_ (i.e. 35–60 mmHg^2^) (see McMorris et al., 2017, 2019). However, although according to more recent research (Angeli et al., 2019), as well as some earlier research (Beidleman et al., 2014; Boos et al., 2016), NH and HH are not the same, more specific data from Hutcheon et al. (2023a) may support McMorris et al.'s (2017, 2019) proposal Indeed, by characterizing the correlations between SpO_2_ and EEG measures in different time spans, Hutcheon et al.'s (2023a) results showed that participants desaturated during the first 150 s of NH, whereas they steadily desaturated during HH. The desaturation period expressed a robust pattern of the aforementioned correlations across frequencies and brain locations. Specifically, the first 150 s of NH during desaturation differed significantly from the HH and NN conditions with negative absolute alpha power-SpO_2_ correlations and positive multiscale entropy (MSE)-SpO_2_ correlations. After steady desaturation, HH had no significant differences in EEG-SpO_2_ correlations.

According to these indications, it is crucial to consider the desaturation phase of hypoxia as a critical period in the course of HH, which would require the development of strategies to mitigate the hypoxic stimulation in a real-life situation.

## Acute mountain sickness and neurocognitive functional changes due to hypoxia

Oxygen is constantly needed by the brain for both normal and most suitable cognitive processes. Even without considering non-traumatic forms of brain injury, alteration of blood flow and oxygenation can have a substantial impact on an individual's mental and cognitive processes (Hopkins and Bigler, 2012).

*High altitude sickness* (HAS) or *Acute Mountain Sickness* (AMS) is a prevalent group of diseases among hikers as more and more individuals climb peaks at very high altitudes, often despite little training. These conditions typically affect mountain climbers at elevations over 2,500 m (~8,202 ft), with implications for the brain and lungs. Acute mountain sickness, or malaise with less severe consequences than the primary kind of *high-altitude cerebral edema* (HACE),^1^ is caused by hypoxia. Symptoms of AMS include headache, fatigue, nausea, vomiting, dizziness, and sleeplessness (Lawley et al., 2014). On the other hand, the consequences of HACE may be more severe and include altered awareness (Fayed et al., 2006). The difference between AMS and *chronic hypoxia condition* (CHC) is that the former is a transient respiratory disruption brought on by extended exposure to low oxygen levels, while the latter is a chronic condition that lasts for several weeks or months if not years (Virués-Ortega et al., 2004; Ma et al., 2015, 2019). The present review will primarily consider investigations of actual ascents as well as conditions of hypoxia that have been created and replicated in the lab.

## Detrimental effects of hypoxia on neurocognitive system

In terms of the main deficits observed, exposure to HA hypoxia can lead to a greater or lesser impairment of most neurocognitive functions, such as reduced processing speed, impaired judgement, expectancy, problem solving, attention and decision making. However, here we will focus on the detrimental effects of hypoxia on cognitive and motor functions related to short-term memory, attention and executive control.

This is because working memory and attention are fundamental cognitive functions that have a significant impact on other processes such as perception and decision making. Highlighting these areas can improve our understanding of the cognitive architecture, as they are deeply interconnected with several other important functions. Furthermore, by emphasizing working memory and attention, the discussion remained clear and focused, thoroughly exploring key processes relevant to the topic without overwhelming the reader.

## Memory

Memory operates at both the cellular/molecular and systemic level. At the cellular level, memories are stored as changes in synaptic connections; long-term memories require more permanent alterations in gene expression, protein synthesis, and new synapse formation. Memory systems typically include three types: working memory, declarative memory, and non-declarative memory. Key distinctions are the duration of memories (short-term vs. long-term) and the quality of information (declarative vs. non-declarative). Declarative memory involves the recollection of events and facts that can be verbally reported, while non-declarative memory is reflected in performance.

One view is that memory systems differ in encoding and retrieval strategies but share cellular storage mechanisms (Squire and Alvarez, 1995; Tulving, 1995). Encoding requires synaptic consolidation, while storage involves transferring memory traces from the hippocampus to the cortex, as per the “standard” model. The “multiple trace” model suggests the interactive creation of episodic and semantic memories in the hippocampus and medial temporal cortex (Hintzman and Block, 1971; Squire et al., 2004). Both models indicate that the hippocampus learns rapidly while the cortex does so more slowly.

Given the diversity of memory systems and the challenges of categorizing them, further discussion is beyond our scope. However, we will focus on some aspects of working memory because of its close interactions with other cognitive functions.

### Working memory

Working memory is a crucial neurocognitive system involved in functions such as perception, long-term memory, attention, language comprehension, and executive functions. It closely interacts with motor systems for executing actions based on processed information. Working memory includes spatial and object feature memory, with spatial information maintenance being essential for processing, navigation, and motor tasks in humans and animals.

It also plays a key role in temporary information processing and storage for tasks such as mental rotation, action planning and decision making. However, exposure to deleterious hypoxic conditions can adversely affect cognitive functions, including attention and episodic memory (Suzin et al., 2020), and reduce overall cognitive resources, as indicated, albeit with some caveats, by the results of a study by Li et al. (2021). This highlights the importance of working memory in supporting cognitive processes and effective functioning.

Using the Morris Water Maze test for mnemonic abilities, murine research has been conducted under hypoxia and normoxia regimens with a threshold set at 5,500 m (~18,045 ft) a. s. l.; it was discovered that at heights varying between this threshold and 6,400 m (~20,998 ft), spatial memory dysfunctions were experienced; more specifically, detrimental effects were observed in speed, distance traveled, and time taken to reach the goal. Performance at the lower height changed more drastically than at the higher one, thus indicating a more serious malfunction in the acquisition of new abilities than in their application or recovery (Virués-Ortega et al., 2004). From short-term memory tasks, in which lists of numbers were learned at different simulated altitudes, indications of short-term memory deficits were obtained at 3,500 m (~11,483 ft) a. s. l. taken as a threshold. However, the data are not sufficient to conclude that hypoxia due to extreme altitudes leads to a permanent deterioration of memory, but deficits and alterations in spatial memory appear to specifically influence encoding rather than retrieval (Virués-Ortega et al., 2004).

In support of this view, 17 mountaineers were tested at 5,350 m (~17,552 ft) using a neuropsychological learning test to assess changes in neurocognitive functions during acclimatization to altitude. The results clearly showed that by extending the time spent at altitudes above 5,350 m to more than 15 days, the response to a memory task was significantly improved. The improvements resulting from acclimatization were more evident in the organization of information than in the storage of information. The authors suggest that inadequate acclimatization has a detrimental effect on short-term mnemonic cognitive function, and that the resulting impairment may be particularly pronounced in the more demanding technical tasks (Pagani et al., 1998).

## Attention

**Figure 1:**
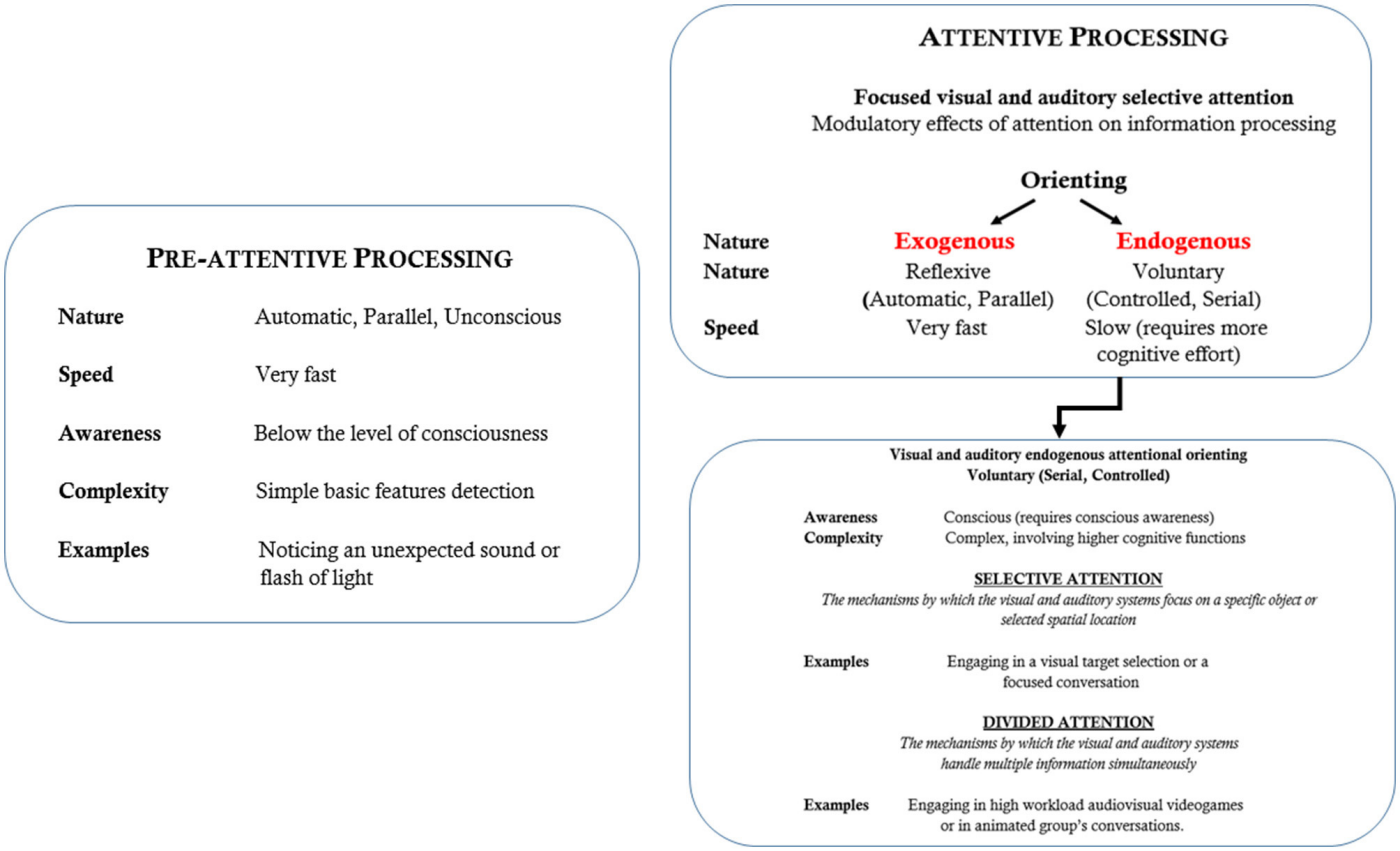
Outline description of the main mechanisms and characteristics of visual and auditory attention.

Attention, as a neurocognitive system, allocates processing resources to pertinent information, filtering out irrelevant data to optimize behavioral responses. Its primary function is to select, prioritize, and activate goal-relevant information while excluding extraneous details that could overwhelm the cognitive system (Posner, 2004). Overall, attention operates through two primary types of information processing mechanisms. The first type of mechanism involves how attention enhances the efficiency of processing relevant information. The second type concerns how our brain and mind control these attentional effects on information processing and execution. The attentional system selects information based on two brain systems: the endogenous (voluntary or top-down) and the exogenous (involuntary or bottom-up) systems (Corbetta et al., 2000; Corbetta and Shulman, 2002). For the second system, various distributed brain areas collaborate to manage attentional resources, aiming to focus on relevant stimuli and spatial locations (e.g., Corbetta et al., 2000). See Figure 1 for a sketched representation of these attentional mechanisms and processing modes.

Just as important as locating and selecting information is dividing attention. Divided attention (see Figure 1 again), as opposed to selective attention, is the ability to simultaneously process multiple signals across different sensory modalities (e.g., haptic, auditory and visual). While both attentional modes manage information overload, divided attention can reduce performance. Foundational studies, begun with dichotic listening tasks (e.g., Broadbent, 1952) showing how individuals process different auditory inputs at once, have expanded our understanding of attention allocation and its implications for cognitive performance in multitasking situations (Marois and Ivanoff, 2005). Visuospatial or spatial attention involves distributing processing power across specific points or regions in space to locate target objects. Access to even the most basic features requires visual attention (Pashler, 1994). Two mechanisms govern spatial attention: a top-down, goal-directed system that is endogenous and voluntary, and a bottom-up, exogenous process that is reflexive and involuntary (Posner, 1978, 2004; Shulman et al., 2004; see Figure 1 again). A substantial body of research has been gathered using the spatial cueing paradigm (Posner, 1978; Corbetta et al., 2000; Corbetta and Shulman, 2002) that demonstrates that targets are detected and well differentiated faster for both voluntary and involuntary attention in validly signaled positions than in invalidly signaled positions (Landau et al., 2007). It is postulated that both types of attention enhance perceptual processing similarly, yet their underlying brain mechanisms are distinct, with differing time courses and outcomes (Posner, 2004). The variations in neural responses between voluntary and involuntary attention conditions support the view that these modalities involve different mechanisms, influencing perceptual processing and performance differently (Corbetta et al., 2000; Corbetta and Shulman, 2002; Landau et al., 2007).

Considering that attention is not a unitary function, the widely accepted view conceptualized by Posner and Petersen (1990) and later by Petersen and Posner (2012) posits that the attentional system is supported by three networks regulating different subprocesses: alerting, orienting, and executive control. In more detail:

- *Alerting*, controlled by the frontal and parietal regions of the right hemisphere, carries out the process of becoming and remaining aware.
- *Orienting* is the process by which the frontal and parietal areas regulate attentional focusing on a given point in space; and,
- *Executive control* is concerned with resolving cognitive conflicts, i.e., a psychological state involving a discrepancy between contingent cognitive information and experience (or between various cognitive mental representations that organize knowledge, beliefs, values, motives, and needs) and psychomotor responses and involves the dorsolateral prefrontal cortex and medial frontal areas (Posner and Petersen, 1990; Petersen and Posner, 2012).

To analyze the functional activation and independence-interdependence of the three networks, an *Attention Network Test* (ANT) was devised by Fan et al. (2002). The reliability of this theory and test has been supported by subsequent studies from the same group (Fan et al., 2005) and several more recent studies, such as that of Markett et al. (2014).

## Research methods for studying selective attention

Like other human cognitive processes, attention is limited by attention-distracting activities or stimuli. Advanced measurement instruments and tests have been developed to reveal the anatomical and functional mechanisms underlying perceptual and cognitive systems. Given the brain's generation of diverse neurophysiological signals, a variety of techniques have been developed to investigate cognitive and attentional information processing from multiple neurofunctional perspectives. They include, overt behavioral responses, i.e., reaction times (RTs) and errors rate (ER), and electrophysiological and electromagnetic methods such as *evoked potentials* (EPs), *event-related potentials* (ERPs) (see e.g., Picton et al., 2000; Zani and Proverbio, 2003; Zani, 2013, 2020) and *event-related magnetic fields* (or ERFs) (Aine and Stephen, 2003; Hari et al., 2018). Measurement technologies related to hemodynamic indices have also been used including *positron emission tomography* (PET) and *functional magnetic resonance imaging* (fMRI). Both ERPs and ERFs have a temporal resolution advantage over hemodynamic neuroimaging techniques, which, on the contrary, have a spatial resolution advantage over the former research tools (Zani et al., 2003). The research markers previously introduced will now be discussed separately, emphasizing their increasing ability to reveal functional mechanisms and the brain's anatomical centers and networks most affected by hypoxia.

## Hypoxia, reaction times, attentional performance, and perceptual discrimination

It is generally renown that hypoxia has an impact on overt reaction times. In fact, one of the most reported effects of altitude and/or oxygen deficiency is the slowing of overt motor RTs, which increases in both lab and real-world contexts. These results were consistently achieved in more recent (e.g., Zani et al., 2020, 2023; Wang et al., 2014, 2021) as well as traditional studies (McFarland, 1932, 1969, 1972; Fowler et al., 1982, 1987). In an attempt to disentangle the effects of acute NH on the sequential processing stages from the perspective of Sternberg's serial processing AFM (i.e., *Additive Factors Method*), in a study by Fowler et al. (1982) in which stimulus features (i.e. two levels of brightness), number of response options (i.e. five response options) and movement distance for key presses were varied, participants were exposed to a low oxygen mixture. Testing was performed with a maximum frequency of one session per day and a minimum frequency of one session per week. During hypoxia, blood oxygen saturation (SaO_2_) was maintained at an average level of 64.3%, equivalent to a HA of 6,700 m (~21,982 ft), for 10 min preceded and followed by air breathing for each of the two-stimulus function runs. The results showed that although overall NH slowed the participants' response time compared to normobaric normoxia (NN), in contrast to more central processes early visual processing stages attained shorter RTs in acute NH than in NN.

A later study by Fowler et al. (1987) used six levels of acute NH caused by low-oxygen air mixtures that reduced arterial oxyhemoglobin saturation (SaO_2_) to values ranging from 86% to 76% in increments of 2%. A separate session was conducted for each level, consisting of two parts and three assessment times. The sessions were 60 min long, 1 day apart, and focused on visual acclimatization before conducting 90-s tests with a fixed error rate. Altitudes between 8,900 ft (~2,713 m) and 11,400 ft (~3,473 m) were represented by the aforementioned levels. Effects of hypoxia caused perceptual-motor function to begin to diminish at altitudes as low as ~9,750 ft (~2,972 m). With an apparent substantial effect at a SaO_2_ of 82% (~10,000 ft; ~3,048 m), response time resulted in a dose-dependent manner being slower. All in all, these results provided a threshold estimate of 9,750 ft (~2,972 m) for performance decrements due to hypoxia and pointed to the disruption of early vision as a factor influencing this decrement.

Preprocessing, feature extraction and identification were some of the earliest perceptual stages that were specifically affected by acute hypoxia, according to additional research by Fowler et al. (1993). These authors observed that hypoxia did not slow down all visual processes, but rather impaired a preprocessing stage related to visual acuity. Lindeis et al. (1996) proposed that the disruption of complex tasks, such as lapses of attention and short-term memory decrements, could be attributed to early slowing acting as a bottleneck to later processing. This suggested that early visual processes played a crucial role in visual performance, although the exact nature of these processes remained unclear based on these findings.

To further investigate the mechanisms subserving the effects of hypoxia on early visual processes, Stivalet et al. (2000) examined the effects of this oxygen-deficiency during a visual search task under different levels of pop-out stimulus conditions. The study used a visual search task to distinguish between preattentive and attentional processes in line with Treisman and Gelade's (1980) *feature integration theory* (FIT) that early visual analysis involves two different modes: automatic or parallel/preattentive and controlled or serial/attentional processing. The experiment involved twenty-one participants, half of whom wore a 5 cm H_2_O positive-end-expiratory pressure (PEEP) device to explore the possible prevention of performance impairment. Each participant was exposed to 8 h of hypoxia in a hypobaric chamber (~14,764 ft, ~4,500 m, 589 hPa, 22°C). During the experiment, the participants' task was to detect a target among distractors in normoxia (i.e., ~722 ft, 220 m; 992 hPa), a test carried out the day before the simulated ascent), in acute NH (i.e., 1 h after the ascent to ~14,764 ft, 4,500 m; 589 hPa), and in prolonged HH (i.e., 7 h after the ascent to ~14,764 ft, ~4,500 m, 589 hPa). Results showed that, unlike short-term acute hypoxia, prolonged hypoxia slowed serial/attentional processes while leaving parallel/preattentive processes unaffected. Participants who were more sensitive to AMS experienced the most significant effects, but PEEP somehow helped to prevent slowing of attentional processing-related RTs in these individuals.

Importantly, Beach and Fowler (1998) also investigated whether the effects of hypoxia concerned, in general, all sensory modalities or were modality dependent, measuring RTs in an AFM paradigm to auditory and kinesthetic stimuli. The results showed that NH [i.e., a 65% SaO_2_, corresponding to a permanence at a HA of ~4,061 m (~13,323 ft) for ~10 min] induced a slowing of RTs to auditory tones but not to kinesthetic stimuli. The authors tried to explain these findings discrepancy advancing the view of modality differences between the AFM bottleneck hypotheses. Furthermore, due to the contradictory findings concerning the effects of hypoxia on auditory thresholds, Fowler and Grant (2000) carried out a study to clarify this issue. Participants breathed either air or a low-oxygen mixture to maintain SaO_2_ at 74% [equivalent to a persistence at ~3,567 m (~11,703 ft) HA], which was stabilized for 5 min prior to the start of the test after a progressive decrease over a period of ~10 min. Hypoxia produced significant statistical effects that resulted in insignificant threshold decrease of only 1 dB with respect to the frequencies administered. To explain their findings, the authors suggested that auditory processing is relatively insensitive to hypoxia, and the observed slowing of responses to auditory stimuli is due to central neural mechanisms rather than increased auditory thresholds. They proposed that the effects of hypoxia in simple reaction time tasks are primarily focused on the early neurofunctional mechanisms of the visual system, although the specifics of these effects remain unclear. In this regard, in an acute normobaric hypoxic bout lasting 4 h at a simulated altitude of 4,200 m (~13,780 ft), Zani et al. (2020) showed delayed RTs in simple choice laboratory visuocognitive cued attentional tasks of both endogenous (or voluntary) and exogenous (or reflexive) type, but not in a double-choice endogenously cued attention orienting task. It is also crucial that the same study discovered that there was a considerable and more pertinent rise in overt motor response mistakes in acute hypoxia in relation to increased motor workload and top-down, endogenous attention allocation as compared to exogenous attention.

It is also important to study differences between factors that may interact with hypoxia or exacerbate its effects. Given the important implications for safety protocols in aviation and transport, particularly with regard to working hours and rest periods of flight personnel such as helicopter pilots in air medical services, an interesting study by Elmenhorst et al. (2009) investigated the differential effects of low to moderate alcohol consumption, 4 days of sleep deprivation and different oxygen levels in acute NH. The oxygen concentrations studied were 13% (~12,000 to 14,000 feet or ~3,658 to 4,267 m), 11.5% (~15,000 to 18,000 feet or ~4572 to 5,486 m) and 10% (~18,000 to 20,000 feet or ~5,486 to 6,096 m) compared to a baseline of 21% at sea level (s. l.). Cognitive performance (including reaction times and lapses of attention) and physical performance (such as unstable tracking tasks) were assessed throughout the study. The results showed that low to moderate alcohol consumption, prolonged sleep deprivation and more severe hypoxia all had comparable detrimental effects on performance. While alcohol increased performance lapses, sleep deprivation had a more significant negative effect on attention than hypoxia. Performance impairment was cumulative over the four sleep deprivation days. Interestingly, one night of 8 h of sleep after sleep deprivation restored performance almost to baseline levels, although the authors report that this latter finding should be treated with caution due to the ongoing training effects throughout the study.

Intriguingly, recent data also support the view that the effects of hypoxia may also depend on the individual's domain-specific cognitive skills in relation to the condition-related safety and survival demands. Indeed, in a study assessing reaction times (RTs), response accuracy, and subjectively rated alertness of professional military helicopter pilots performing a visual choice reaction task that corresponded to eye-tracked attentional scanning of helmet-mounted display (HMD) symbology, Steinman et al. (2023) found that HH (a simulated ascent to 4,572 m (~15,000 ft) progressing in five equivalent steps compared to 92 m (~300 ft) in a hypobaric chamber), during which two 20-min periods of active performance were experienced, decreased apparent reaction speed (i.e., increased reaction time) while increasing the accuracy of these pilots' responses to visual stimuli presented in low and high contrast and in different fields of view (FoV), namely 30 and 50 degrees FoV. Interestingly, reduced subjective alertness did not affect response accuracy. All in all, then, it seemed that pilots exerted more information processing effort in hypoxia to keep steady performance levels.

On the basis of the above data, it may be assumed that exposure to HA affects the brain and cognitive processing functions, leading to a slowing of overt responses to sensory stimuli, apparently starting at a SaO_2_ of 82%, roughly corresponding to a HA of 3,048 m (~10,000 ft) a. s. l. Consistent with this proposition might also be the recent scientific discovery of the Baishiya Karst Cave, located on a steep rocky slope on the Tibetan Plateau at a roughly analogous altitude of 10,700 feet (3,261 m), where oxygen begins to be noticeably reduced and the climate is cold and dry. In fact, a number of archaeological expeditions to the cave in recent years have shown that it was home to one of the most mysterious branches of humanity, the Denisovans, who thrived in this harsh climate for over 100,000 years, successfully adapting to the Tibetan Plateau even during the Ice Age, which says something about their resilience and the hospitality of that environment (Xia et al., 2024).

It may also be assumed that an individual's specific cognitive skills and condition demands influence the effects of hypoxia on cognitive processing. However, understanding how these effects impact processing stages is not directly accessible through behavioral measures.

## EEG and brain investigation techniques

**Figure 2:**
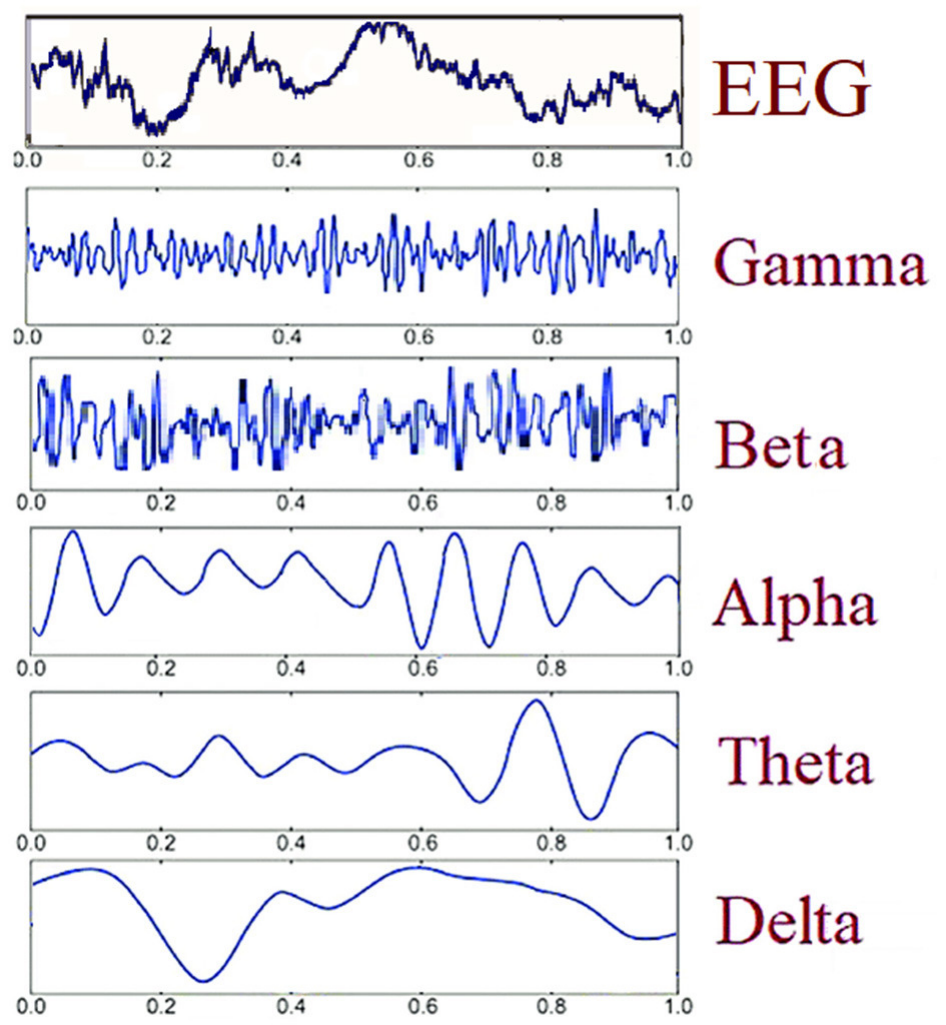
Schematic representation of different temporal frequencies and amplitudes for EEG oscillations.

It is well established that the level of oxygen in the blood affects the electrical activity of the brain. This view is based on several studies that have identified differences in the EEG activity spectrum, such as increases in delta and theta oscillations (e.g., Wang et al., 2021) and fluctuations in alpha power, both at rest and during active psychomotor tasks (Zani et al., 2020). Neuro-electrical activity is directly impacted by hypoxia, and this can result in deficits in higher-order cognitive functions and sensory perception. This is because proper brain activity necessitates an adequate amount of oxygen (see Wong et al., 2023 for a very interesting review on this subject). EEG is one of the main methods to study brain activity by recording the macroscopic temporal dynamics of the brain's electrical activity bands using scalp sensors (or electrodes; Zani and Proverbio, 2003; Rippon, 2006; Proverbio and Zani, 2013; Zani, 2013; Chiarelli et al., 2017). Considering the differences in the literature, the following categorization of EEG bands may be appropriate: Delta: 0.5–4 Hz, Theta: 4–7.5 Hz, Alpha: 8–13 Hz, Beta: 14–30 Hz, Gamma: > 30 Hz. These bands are not rigidly defined and vary with the individual's state of rest or activity (see Figure 2). Recent subdivisions include slow and fast alpha (7–11 and 12–14 Hz) and multiple beta bands (beta 1, 2, and 3) going from 14–16, 17–20, and 20 Hz and above, respectively. Distinct brain regions exhibit specific rhythms, such as tau in the auditory cortex and mu in the somatosensory cortex. Amplitude or power specifies frequency activity, with typical ranges like alpha (25–100 μV) and beta (under 20 μV).

The most used methods for extracting the indicated frequency bands from raw EEG are the fast Fourier transform (FFT) and wavelet, but other methods are also used, such as the Short-Time Fourier Transform. The unit of measurement in the power spectrum or power spectral density is very often μV^2^/Hz and the result of the wavelet analysis is often expressed in dB.

The oscillation bands derived from EEG recordings and deconvolutions are associated with distinct functions of the various brain regions, especially those related to associative processes (Başar et al., 2000). Thus, integrative activities at all sensory and cognitive levels are controlled by the delta, gamma, theta, and alpha oscillatory systems (Başar et al., 2001). The information indicates that oscillations are linked to brain structures, tasks, and sensations (Başar et al., 2000, 2001; Psatta and Matei, 2021). Extensive studies on EEG, ERPs, and MEG rhythms were conducted during the “Decade of the Brain” (1990–2000). Notably, it was found that the brain's response to auditory and visual stimuli in the reticular formation, hippocampal regions, and audio-visual modules correlates with the alpha rhythm range (Popov et al., 2017). Additionally, alpha oscillations have been associated with both short- and long-term working memory. Generally, the alpha rhythm was linked to a variety of sensory and cognitive processes. The theta rhythm was evoked in response to bimodal sensory stimulation, suggesting that complex events cause electrical activity in the 4–7 Hz range. Changes have been observed in the limbic system and frontal, prefrontal, and parietal cortex because of EEG and ERPs derived from paradigms that induce focused attention. Results from certain trials with cats during exploration and search behavior imply that this rhythm is related with the orienting system, from which a coordinated response indicating attentiveness or arousal directed at information processing is coming (Başar et al., 2000, 2001).

## Hypoxic effects on rhythmical EEG markers of vigilance and attentional processes

Research into hypoxia reveals a complex relationship with EEG signals. The challenges in comparing EEG studies that examine hypoxia arise primarily from significant variability in hypoxic stimulation, which can differ in both intensity and duration. Additionally, factors such as experimental design disparities, particularly between normobaric and hypobaric conditions, add further complexity to the analysis. Compounding these issues is the evolution of EEG technology; advancements unavailable in earlier research can lead to skewed interpretations of results.

An important factor that the researchers considered was how hypoxia affected the EEG signals differently depending on whether the participants had their eyes open or closed. In instances where eyes are open, increased alpha frequency band power (8–12 Hz) is typically observed during hypoxic episodes. Conversely, when participants close their eyes, this alpha power generally diminishes (Schellart and Reits, 2001; Kraaier et al., 1988; Ozaki et al., 1995; Papadelis et al., 2007). Such behavior contrasts with normoxic scenarios, where eye state changes yield more predictable patterns in alpha activity (Barry et al., 2007).

Despite the preponderance of these findings in the literature, the distinction between the increase in resting alpha and the decrease in resting signal power is somewhat contradicted by some recent studies. The first, by Zani et al. (2020), used four different visuospatial cueing attention tasks of increasing difficulty during an acute 4-h normobaric hypoxic episode, during which alpha power increased significantly. In contrast, using a simple visuospatial task Hutcheon et al. (2023b) found a decrease in alpha during short 25 min bouts of normobaric hypoxia.

### How hypoxia affects bands other than the alpha band

Besides alpha, research findings regarding hypoxia often indicate enhancements in various other frequency bands, including delta, theta, and beta (Kraaier et al., 1988; Schellart and Reits, 2001; Schneider and Strüder, 2009). However, some studies offer a contradictory perspective, showing significant reductions in the power of theta, alpha, beta, and gamma waves during hypoxia (Rice et al., 2019a,b). Moreover, analyses at the source space level have shown an uptick in low beta power (12.5–18 Hz) in the right superior frontal gyrus during mild hypoxic conditions (Schneider and Strüder, 2009).

The discrepancies observed in these research outcomes can, in part, be attributed to differences in methodology, including the use of dry-EEG systems that mitigate electrical noise by isolating each sensor within its own Faraday cage. Furthermore, modern studies often employ larger sample sizes compared to their predecessors, a factor that likely influences the variation in research findings.

### Hypoxia, hypocapnia and gamma band

EEG gamma oscillations are specifically induced by visual stimuli and increase with selective attention demands. Intriguingly, gamma oscillations have also been actively investigated in conditions of hypocapnia. This alteration, in fact, is not only caused by exposure to HA, but also voluntarily, as in the case of hyperventilation (HV), which is also a consequence of excessive breathing in hypoxia that reduces the partial pressure of CO_2_ (i.e., hypocapnia), leading to respiratory alkalosis. Hypocapnia leads to alterations in physiological and cognitive function and, in extreme cases, can cause convulsions in susceptible individuals and epileptiform brain activity on the EEG. In a study by Stenkamp et al. (2001) on a sample of human volunteers, it was found that in six out of eight participants, VEPs were constrained by the gamma band (in this case between 30 and 40 Hz) and the gamma rhythm increased during voluntary hyperventilation. Moreover, Jensen et al. (2002) investigated the impact of hyperventilation on phase-locked oscillatory responses in the human brain. They measured visually evoked magnetoencephalographic responses before, during, and after hyperventilation using checkerboard stimuli, revealing enhanced gamma-band (30–45 Hz) responses in the occipital cortex that maintained frequency stability. A neural network simulation showed heightened spontaneous gamma activity in rats' hippocampus during hypocapnia, linked to increased GABA levels. The authors suggested physiological mechanisms involving GABA-A receptor inhibition and neuronal excitability to explain their results. Interestingly, several studies have shown that hypocapnia can improve neuronal excitability even if synaptic transmissions are altered, and data suggest that evoked gamma activity plays an important role in early sensory processing.

## ERP markers of effects of hypoxia on cognitive and brain functions

### EPs/ERPs markers and hypoxia

**Figure 3:**
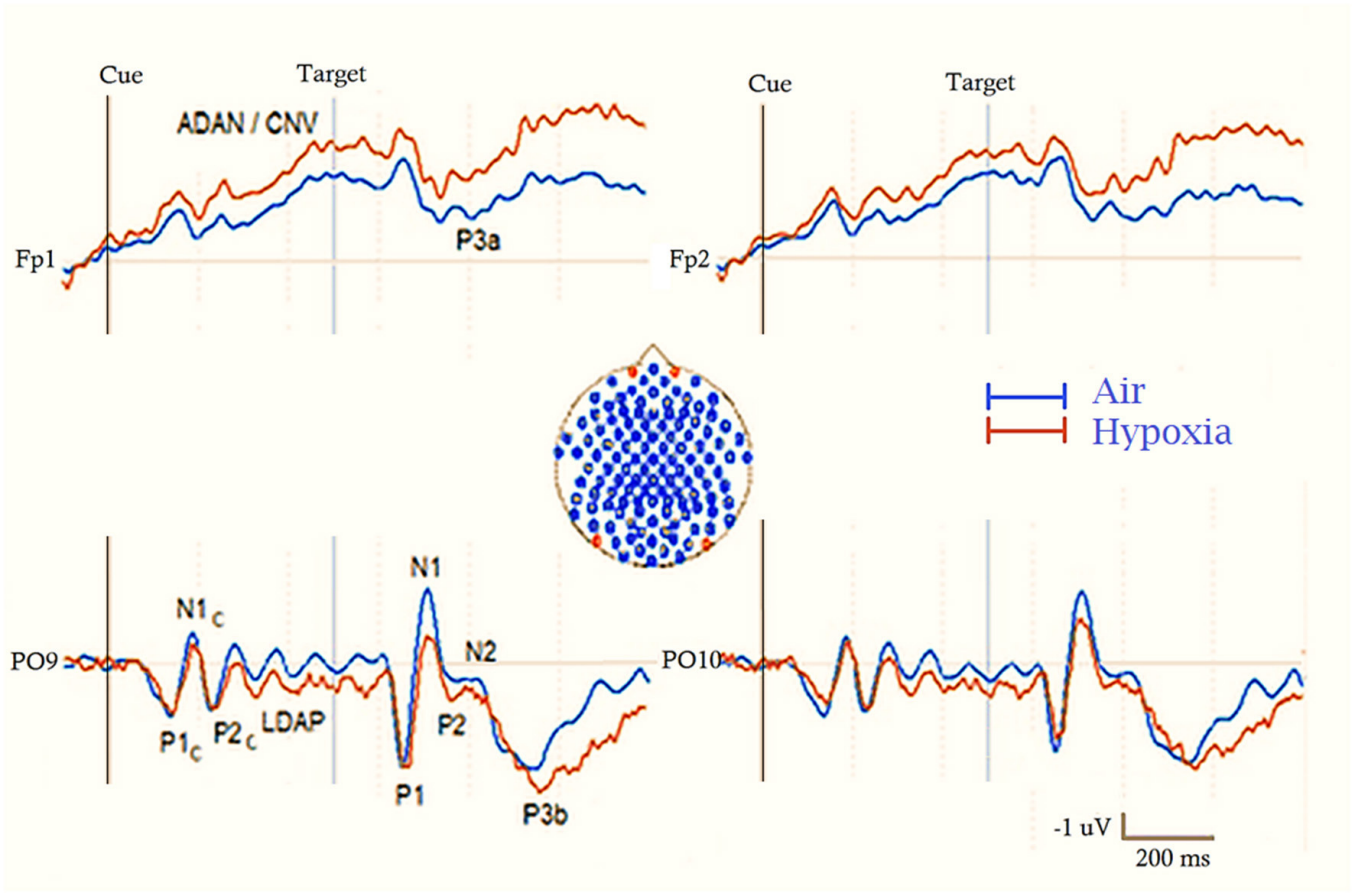
Schematic drawing of ERP waveforms recorded during a cue (Cue) and target (Tgt) spatial attention orienting task at prefrontal and occipital electrodes (at locations indicated by the red discs in the inset at the center of the figure). The two superimposed lines indicate the brain response to ambient air (in blue) and acute hypoxia (in red) respiratory conditions, respectively. Cues induce early P1c, N1c and P2c components, followed by a prominent Late Directing Attention Positivity (LDAP) at posterior scalp sites and a prominent Anterior Directing Attention Negativity/Contingent Negative Variation (ADAN/CNV) at anterior sites. Targets, on the other hand, elicit the pool of sensory-perceptual components seen for the cue at parietal-occipital scalp sites, followed, depending on the experimental paradigm, by either a pronounced fronto-central P3a in a novelty detection task or a more pronounced parieto-central P3b in an ordinary different semantic processing task.

Besides EEG bands *evoked potentials* (EPs) and/or *event-related potentials* (ERPs), i.e., the consistent variations in ongoing electrical activity that are brought either by the features of the direct stimulation or the contextual cognitive processing that control these variations (Picton et al., 2000; Zani, 2013, 2020), have been, and are still fruitfully used, to study the possible effects of hypoxia on human mental states such as memory, expectancy, vigilance or different stages of attentional processing (Wong et al., 2023) (see Figures 1, 3).

### ERP P3s and the oddball paradigm

Although many cognitive domains are explored through various experimental paradigms, the “oddball paradigm” remains the most prevalent for investigating how hypoxia affects electrophysiological markers of selective human information processing (HIP). In this task, participants are required to respond quickly and accurately to a sequence of stimuli, where one (the “standard”) appears more frequently than the other (the so called “deviant” or target stimulus). Participants can be instructed to respond to stimuli in a variety of ways, for example by pressing a reaction time button or by silently counting in motorically passive oddball modes. However, button pressing is most used to investigate the relationships between the covert neurofunctional stages of information processing and the overt psychomotor response stages (e.g. Kutas et al., 1977). From an electrophysiological point of view, in addition to previous early latency sensory-perceptual responses, the rare target elicits a late latency positive-going response reflecting a “semantic” processing of this stimulus, defined as P3b and having a parietal-frontal scalp topographic distribution, occurring around 300 ms depending on the stimulus condition and task demands.

In a three-stimulus oddball version, a third, rarer stimulus is introduced to investigate responses to “novelty,” reflected by a so-called P3a component, which has a larger reversed frontal-parietal scalp distribution (e.g. Squires et al., 1975). These components are typically used to investigate how brain activity relates to task complexity and the timing of mental processing. However, since the definition of the P3 is not uniform in the literature, besides the indicated components, a P3 “family” must be considered referring to the P3a/novelty, P3, P3b, no-go P3 components, while in other cases P3 refers to P3b. Hence, it is especially difficult to find out what P300 means where the task/type of stimulus is not described.

### In search of EEG dipole localization of hypoxia effects

The localization of reversible and/or persistent effects in the brain have also been under investigation, although this has been the object of a scant number of studies. Indeed, by recording scalp electrical potentials, only a few studies have used source analysis algorithms to identify the intracerebral electrical sources of these brain functional effects and impairments. No doubt, BESA (or *Brain Electrical Source Analysis*; Scherg, 1992) and LORETA (or *Low-Resolution Electromagnetic Brain Tomography*) are the most efficient methods to precisely identify 3D intracranial peak activity areas with little “blurring” (Zanow and Knösche, 2004).

## ERP markers of hypoxia effects on attention and memory

The impact of hypoxia on neurocognitive functions has been extensively studied using ERP markers. Indeed, as numerous ERP investigations have demonstrated (Dobashi et al., 2016), hypoxia affects manifold ERP components related to working memory (Malle et al., 2013), attentive processing, and overt motor responsiveness (Wang et al., 2014, 2021).

However, large methodological diversities between studies, including different methods used to induce acute hypoxia (i.e., normobaric vs. hypobaric), differences in altitude (in between ~3,000 m; ~9,843 ft–~6,400 m; 20,998 ft), duration of hypoxic exposure (i.e., acute, short-term, long-term and/or chronic hypoxia), time span in case of the use of acute mode, subjects' variability, task used, different pre-attentional and attentional-related distributed information-processing stages, and, in relation to this, the manifold ERP components considered, make it difficult to draw definitive conclusions about the nature of the detrimental effects of hypoxia on brain anatomy and neurocognitive functions.

This is partly due to the scarcity of studies examining focal and distributed intracerebral activations and deactivations through hemodynamic measures or brain source reconstruction of electrophysiological data, compared to the extensive research on the general effects of hypoxia on cognitive, physiological, and health functions. Additionally, existing studies are categorized into hypobaric and normobaric hypoxia investigations, as well as “resting state” and “task activation” studies, which may cover various cognitive domains and associated brain areas and networks. Where information processing and selective attention are concerned, most of ERP studies in the literature dealt with the influences and modulations by hypoxia on mechanisms undergirding selective processing of relevant sensory input. Conversely, only a very little recent studies have focused on neural processes and mechanisms by which these modulatory effects on stimulus processing are regulated.

### Effects of acute hypoxia on ERPs related to preattentive processing

Preattentive processing refers to the brain's ability to automatically detect and rapidly assess incoming sensory stimuli for significance without requiring focused attention. It generally involves components such as auditory Mismatch Negativity (MMN) that can be recorded also during sleep. Early and perceptual components such as the C1 (60–80 ms) and P1 (80–120 ms) visual responses, reflect rapid, automatic responses to sensory stimuli, indicating initial detection in the visual cortex, but are largely modulated by attention. For example, the visual N40 (around 40 ms) serves a critical role in early visual processing, suggesting thalamic involvement and capturing rapid responses to visual stimuli, reflecting attentional selection (Proverbio et al., 2021). Later ERPs such as the MMN (100–250 ms) reflect automatic detection of deviations in auditory stimuli, while the visual MMN serves a similar function for visual changes. The P3a (250–350 ms) marks the transition to exogenous attentive processing, indicating attentive engagement and resources allocation in response to unexpected stimuli. Together, these components highlight the spectrum between immediate sensory detection and more elaborate cognitive processing, emphasizing the brain's efficiency in handling sensory information.

In this respect, early ERP-based attention studies indicated that acute hypoxia generally did not affect the earliest stages of perceptual processing (e.g., C1, P1, N1, P2, and N2 components; Fowler and Lindeis, 1992; Kida and Imai, 1993; Wesensten et al., 1993; Hayashi et al., 2005). These findings have been consistently reconfirmed in subsequent research. However, recent studies have reported detrimental effects of hypoxia on preattentive and automatic processing in both visual and auditory modalities, providing objective, time-based support for the behavioral findings of Fowler et al. (1987), Beach and Fowler (1998), and Stivalet et al. (2000). In a study by Seech et al. (2020), for instance, the ability to recognize novelty in the surroundings was explicitly investigated utilizing the so-called *auditory deviation response* (ADR), which is generated in the absence of explicit attention and directs the functionally appropriate neural networks for testing. The ADR is composed of three peaks: “novelty P300,” also defined as *P3a*, according to Squires et al. (1975), *mismatch negativity* (or MMN; see Näätänen et al., 2003 and Brattico et al., 2013 for a description of this component functional properties), and *reorientation negativity* (or RON; see Higuchi et al., 2014 on this ERPs response), a negativity that reflects attentive reorientation back toward a task after distraction. Using an auditory paradigm and a continuous visuomotor monitoring activity, these passively induced electrophysiological measures, defined “preattentive” auditory information by the authors, Seech et al. (2020) revealed a significant decrease in the amplitude of P3a during the hypoxic condition (i.e., 10.6% O_2_, i.e., ecological equivalent of ~17,500 ft, ~5334 m) compared to the normoxia condition (20.4% O_2_). Interestingly, this decrease was already noticeable during the first 9 min of hypoxia exposure and affected behavioral task performance. In contrast to P3a, no hypoxic impacts were found on the MMN and RON components. The P3a, a passively elicited brain measure of neural processing, has therefore been indicated as a meaningful and helpful biomarker for demonstrating that the brain is vulnerable to hypoxia, in accordance with the conclusions drawn by the authors based on these findings.

Regarding significant effects of acute normobaric hypoxia on visual information processing as reflected by ERPs, very interesting findings were reported by Altbäcker et al. (2019) in a sample of participants performing three-way oddball tasks under normobaric hypoxia of 80% SaO_2_, reached in gradual steps, namely, from normoxia to 90% in 6 min, a permanence at 90% for 10 min, then a decrease from 90% to 80% SpO_2_ in 7 min, with a staying at this level for 20 min and a going back to normoxia in 7 min. The authors found that acute normobaric hypoxia induced by the indicated modality [corresponding to 5,500 m (~18,045 ft) a. s. l.], had no effects on the efficiency of a visual task-relevant, target-related Go P3b or a target-irrelevant No-Go P3b as well as on behavioral efficacy, but it significantly decreased the efficiency of a novelty-related P3a. The authors concluded that the inconsistency between the trend of the former prefrontal components and the parietally distributed late latency response argued against the view that these responses were variants of the same component, and, overall, that hypoxia disrupted a more specific sub-process of apparent prefrontal origin.

More recently, Blacker et al. (2021) investigated the effect of hypoxia on *visual mismatch negativity* (vMMN). Like its auditory counterpart, this ERPs component is automatically elicited by visual stimuli that differ from predefined patterns based on prior events (vMMN) and, according to the influential review by Kremlaček et al. (2016), is thought to represent an automated prediction error response generated by brain systems that build probabilistic representations of sensory inputs. In contrast to what was found for the MMN in previous studies, the amplitude of the vMMN component decreased significantly by 50% during a bout of normobaric hypoxia, during which, analogously to Seech et al. (2020), participants' oxygen concentration was reduced from 20.4 to 10.6%, measured at sea level, simulating an altitude of ~17,500 ft (5,334 m). The reviewed data indicate that acute hypoxia differentially affects various cognitive states, including behavioral tasks and performance, reaction times, errors, perceptual sensitivity, and higher-order cognitive abilities.

### Effects of acute hypoxia on late-latency ERPs

In the context of acute hypoxia, pioneering ERP attention studies have generally found that the N1, P2, and N2 components remained unaffected. However, hypoxia significantly modulated late latency components such as P3b, which is associated with attention and conscious perception, and Contingent Negative Variation (CNV), a slow negative brain wave occurring between a warning stimulus and an imperative stimulus in S1–S2 paradigms. Both components are primarily linked to attention and alertness. In this regard, both P3b latency and behavioral performance have been reported to be affected and significantly delayed by hypoxic conditions in oddball paradigms (Kida and Imai, 1993; Wesensten et al., 1993; Hayashi et al., 2005). Importantly, Hayashi et al. (2005) showed how hypoxia affected P3b latency more than hypobaric conditions, as oxygen administration counteracted the effects of simulated hypobaric altitude. In addition, both a CNV-like frontal negative slow wave and a parietal positive slow wave were found to progressively increase in amplitude with increasing hypobaric hypoxia (Kida and Imai, 1993). Interestingly, while slow waves in the ERPs were observed in participants with delayed reaction times at HA, these waves either diminished or disappeared when participants failed the go/no-go reaction time task. This suggests that these slow waves may be linked to efforts to sustain behavioral responses despite the increasing workload effects of hypoxia. However, a later study by Takagi and Watanabe (1999) on the acute effects of hypobaric hypoxic conditions in a S1–S2 stimulus paradigm with a 2 s interstimulus interval found that the amplitude of a late CNV (l-CNV) showed a negative correlation with RTs at altitudes of 3,000 m (i. e, 9,843 ft) and 0 m. Conversely, at higher altitudes of 4,000 m (i.e., 13,123 ft) and above, this negative correlation concerned the amplitude of an early CNV (e-CNV), although at 6,000 m (i.e. 19,685 ft) the amplitude of the whole slow-wave was greatly reduced compared with that at sea level (or 0 m).

More recent ERP studies, involving a variety of tasks, reported various effects of hypoxia. For instance, Tsarouchas et al. (2008) recorded ERPs to visual targets and non-targets in an ultra-rapid categorical discrimination task under the conditions of normoxia and moderate hypobaric hypoxia, namely the rising to 15,000 ft (~4,572 m) height in 5 min. The data revealed attenuation of early P1 but enhancement of long-latency N1–P3 amplitudes as well as delay of mesogenous P2 latency for both kinds of stimuli. Consistently with previous studies hypoxia also delayed P3b latency and overt motor response times, but only for non-targets.

Blacker and McHail (2022) used a S1–S2 paired clicks paradigm to study the effects of normobaric hypoxia on early sensory evoked potentials. They evaluated visual (P1) and auditory potentials (P50, N1, P2) in healthy adults during normobaric normoxia (NN; 21% O_2_) and exposure to normobaric hypoxia (NH) for 14.5 min (9.7% O_2_, corresponding to a permanence at a height of ~19,124 ft; ~5,828 m). The results showed no significant drop in P1 amplitude under hypoxia, indicating preserved early visual processing. While auditory gating for P50 and N1 was intact, the P2 ratio declined, pointing to impaired attentional processing. Thus, early sensory processing remained unaffected, but auditory attentional processing was compromised. This differs from previous findings of visual performance deficits in hypoxia, suggesting these are not related to early visual processing.

Moreover, Qingguo et al. (2016) found that exposure to transient moderate hypoxia, simulating 5,000 m (i.e., 16,404 ft) altitude, while performing a mental rotation task of two-dimensional letters presented in both normal and mirrored orientations, significantly increased the amplitude of both the ERPs P3b and a slow posterior negative deflection, defined as *rotation-related negativity* [or RRN; see Riečanský et al. (2013) for info on this component]. In Qingguo et al. (2016), the angle effect on the amplitude of RRN was more evident with normal than mirrored letters in hypoxia than in normoxia; interestingly, a more bilateral scalp parietal activation was also reported during hypoxia than normoxia.

## Effects of long-term and chronic hypoxia on ERP components

As with the acute effects of hypoxia, we discuss the preattentive and attentive effects of prolonged and chronic hypoxia separately.

### Effects of long-term and chronic hypoxia on ERPs related to perceptual processing

Regarding the effects of long-term and chronic exposure to hypoxia, studies in lowlanders (i.e. participants who had always lived at sea level) using standard auditory oddball paradigms showed that the latencies of the perceptual components of the ERPs (e.g., N1, P2 and N2), were not affected by hypobaric hypoxia, either on the 6th day of exposure at 3,200 m (i.e., 10,499 ft) or after exposure to 4,300 m (i.e. 14,108 ft) HA on the 8th day of exposure at this altitude (Singh et al., 2004). A similar lack of effect of hypoxia on N1, P2 and N2 components after 1 month and/or after 6 months at 4,115 m (i.e. 13,501 ft) was also reported by Thakur et al.'s (2011) auditory oddball study.

### Effects of prolonged and chronic hypoxia on late latency ERPs

The effects of hypoxia on cognitive ERP components have also been investigated. As far as the classical attention-related P3b component is concerned, significant findings have been reported in the above-mentioned studies. In this regard, the study by Singh et al. (2004) on lowlanders showed that target-related P3b latency recorded on day 6 of exposure to 3,200 m (i.e. 10,499 ft) HA was delayed in only 50% of participants, whereas after exposure to 4,300 m (i.e. 14,108 ft) HA for 8 days, all participants showed an increase in P3b latency. In addition, findings of delayed P3b latency after 1 month and progressively further delay after 6 months of exposure at 4115 m (i.e. 13,501 ft) were reported by Thakur et al.'s (2011).

Importantly, the recording of ERPs and behavioral responses in two groups of participants (Wang et al., 2014) who had either lived at HA (i.e., ~3,650 m; ~11,975 ft) for 3 years with a return to low altitude (LA; i.e., ~884 m (~2,897 ft) for <2.536 ± 0.92 months per year) or LA (i.e., ~621 m; ~2,037 ft) for the same period, showed HA effects on target-related sensory and cognitive neural processing in a cued spatial attention task under high vs. low perceptual load conditions. The HA group showed to be slower than LA group, and to be characterized by an enhanced bilateral parietal N1, and a reduced P3b amplitude to targets under high perceptual load condition. The authors concluded that chronic exposure to hypoxia induced compensation at early processing stages while reducing allocation of spatial attentional resources at later processing stages in relation to the perceptual load.

Zhang et al. (2018) conducted a study to compare the performance of people living at sea level and people who had lived in HA areas (3,680 m; 12,073 ft) for 3 years using a visual search task. The aim of the study was to address the question of whether the slower reaction times to attentionally demanding tasks resulting from chronic exposure to HA depend on changes in attentional allocation and/or response patterns.

In addition to overt RTs, response-related (Motor Potentials, MP, and Reafferent Potentials, RAP) and attention-related (N2 recorded at posterior-contralateral scalp sites, N2pc, and N2 at central-contralateral scalp sites, N2 cc) brain ERPs were recorded. The participants who had lived at high altitude showed significantly longer reaction times than those at sea level when responding to the targets of the visual search test.

These scientists discovered that the HA participants had a much bigger N2 cc amplitude and a significantly smaller N2pc amplitude, which is consistent with these behavioral findings. These results led the authors to propose that these individuals' spatial attention allocation to the target (N2pc) be reduced, implying that they needed to exert more effort to prevent response selection and attention direction (N2 cc) from colliding.

To shed light on the function of altitude in adult *behavioral inhibitory control* (BIC) and the neural mechanisms that underlie it, Wang et al. (2021) examined the neural activity profiles associated with BIC in healthy immigrants who moved from low altitude (LA) regions to high-altitude (HA) regions (3,680 m; 12,073 ft) over a minimum of 3 years. This study used a two-choice oddball paradigm to track the ERPs N2 and P3b, theta and delta band powers, and behavioral reactions in both the LA and HA groups. The HA group had longer response times (RTs) than the LA group, according to the results. Lower N2 and P3b amplitudes were also noted for the HA group in comparison to the LA group. Additionally, significant positive associations between P3b amplitude and theta/delta were found. Overall, this study's neurocognitive findings imply that very long-term exposure to HA may lower BIC during the response inhibition stage.

Using the same two-choice oddball paradigm as Wang et al. (2021), but analyzing different ERPs components, Ma et al. (2015) found greater error-related negativity [ERN; see Gehring et al. (1993, 2012) for extensive reviews of functional accounts of this component] and correct-related negativity [CRN; see Ford (1999) for descriptive attributes of this ERPs component] in the HA than in the sea-level group engaged in a go/no-go task.

Interestingly, long-term/chronic exposure to hypoxia was also found to robustly affect conflict control functions as assessed by ERPs to congruent and incongruent target stimuli during a flanker task in three separate groups of young healthy Tibetans living since birth at an altitude of 2,700 m (i.e., 8,858 ft), 3,700 m (i.e., 12,139 ft), and 4,500 m (i.e., 14,764 ft), respectively (Ma et al., 2019). Despite the lack of differences in behavioral response speed between the groups, the N2 difference wave of the ERPs of incongruent minus congruent stimuli for the three groups showed a significant modulation by cognitive conflict, being smaller in the 4,500 m group than in the other two lower altitude groups.

Taken together, the results reviewed above suggest that the effects of hypoxia (either normobaric or hypobaric) on the neural processing of relevant stimuli falling at an attended spatial location and on psychomotor performance may be task-, altitude- and duration-dependent, and that the thresholds for these effects may be around 3,000 m for at least 1 week.

### Hypoxia affects the neural systems responsible for controlling modulatory functions in selective processing

In contrast to all the above studies, which have investigated whether and how hypoxia affects neural excitation related to sensory and cognitive processing of target stimuli, a particularly useful approach for distinguishing brain activity specifically related to target processing from activity related to higher-systems level processing of preparatory biases in hypoxia is a cued attentional event-related design. Indeed, an advantage of the cueing paradigm for studying attentional control is that it separates the time when a target appears (and when hypoxia may affect its processing) from the time when individuals prepare to focus on a sensory feature or location, based on the cue. Electrophysiological signals, with their high temporal resolution, are ideal for examining the timing of cue-related vs. target-related processes in hypoxia both as oscillatory (non-phase locked, e.g., Zani et al., 2020; Hutcheon et al., 2023b), or pattern-onset (phase-locked, e.g., Zani et al., 2023) signals. Notably, both studies explored the neural sources behind scalp responses to hypoxia.

In these sorts of searches Zani et al. (2020) used a spatial attentional orienting paradigm that relied on four different modalities of endogenous and exogenous cues that were randomly administered. The task was to discriminate two arrow targets of very slightly different shapes, surrounded by congruent and incongruent flankers. The participants had to make either a single or a double choice overt motor response to these target arrows under different runs. The authors found that, overall, 4 h of normobaric hypoxia [NH; achieved by administering a 12.5% O_2_-poor air mixture simulating a constant altitude of ~4,200 m (~13,780 ft) at sea level] significantly increased alpha power in right parietal-occipital and left prefrontal scalp regions compared to the same time span of EEG recorded in NN, irrespective of the attentional cueing condition.

More recently, (Hutcheon et al., 2023b) examined physiological data in conjunction with induced and evoked alpha and beta powers while participants completed an endogenous valid and invalid spatial attention orienting task under control (NN), NH (participation of inspired oxygen = 87.2 mmHg), and HH (3,962 m; ~12,999 ft) conditions in a hypobaric chamber. The three conditions lasted 25 min each, separated by at least 48 h. These researchers found that induced alpha power was significantly reduced in NH and HH than in NN.

It's also interesting to note that, unlike NH and NN, participants in HH had significantly higher evoked beta and induced lower-alpha power, suggesting that NH and HH have different effects on the neurophysiological activity that underlies cognition. As NH and HH could be distinguished by overall EEG spectrum, the authors concluded that they were not neurophysiologically comparable.

Given the influential literature on the relationship between spatial attentional orienting and alpha power, the reported inconsistencies in changes in alpha (either increasing or decreasing) may be attributable to methodological differences among studies. Indeed, a decrease in alpha power has been found in occipital-parietal areas during attentional orienting tasks, whereas an increase in this index has been found during attentional maintenance (Rihs et al., 2009). Further data also indicate that task difficulty and visual stimulus handling can alter alpha band amplitudes in different ways and at different times during cued processing. Participants in the study had to decide whether an object was a car or a face; alpha activity showed higher power in right occipital-parietal regions when the decision task was made more difficult (Li et al., 2013). Given that the effects of hypoxia are known to be closely intertwined with the mechanisms of the cognitive domain under consideration, it seems plausible that the greater demand for stimulus discrimination and response decision required in our attentional control study compared to the work of Hutcheon et al. (2023b) is consistent with the trend of increased alpha in hypoxia proposed by the cited studies in the general literature.

As for the relationship between Hypoxia and ERP components in between cue and target visual stimuli, Zani et al. (2023) recently investigated how participants' brain reacted to hypoxia in four attentional cueing conditions in which the cue could be uninformative because it was completely absent, alerting but not spatially informative, and alerting and spatially informative during preparation for a single/double choice motor response. ERPs were obtained in two randomly administered sessions in which participants breathed either ambient air or oxygen depleted air (12.5% O_2_-poor air mixture corresponding to a HA of ~4,200 m; ~13,780 ft at sea level, or NH) for 4 h. Advanced source reconstructions were also computed. Between the cue and target time span, both an Anterior Directing Attention Negativity (ADAN)/CNV and a Posterior Directing Attention Positivity (LDAP)/TP were recorded, which were larger in ambient air for the valid spatial orienting conditions than for the alerting and uncued conditions. In NH, the amplitude of these two potentials was higher in the valid spatial orientation conditions for the upper visual hemifield, whereas for the lower hemifield, ADAN/CNV largely increased but LDAP/TP decreased for these same attention conditions. This further supported the proposed existence of an anisotropy in the visual field representation in favor of the upper visual field. These ERP changes corresponded to compensatory increased source activations of the right anterior cingulate cortex, left superior parietal lobule and frontal gyrus, as well as detrimental effects of NH on overt behavioral performance.

Taken together, these ERP results seem to indicate for the first time that NH, compared to ambient air, increases inverted polarity changes in activations already during the cue-to-target period and selectively alters visuospatial attentional orientation in space, most likely due to a switch from a more effortless and automatic to a more effortful and controlled information processing.

## Localizing hypoxia effects in the brain: a critical comparative analysis of hemodynamic and bioelectrical markers

As we have seen, oxygen deficiency or starvation primarily damages and affects brain tissue. This has significant psychophysiological consequences. Indeed, when neurons lack oxygen, they catabolize, producing more lactic acid and other catabolic products that damage the neurons irreversibly and eventually kill them.

**Figure 4:**
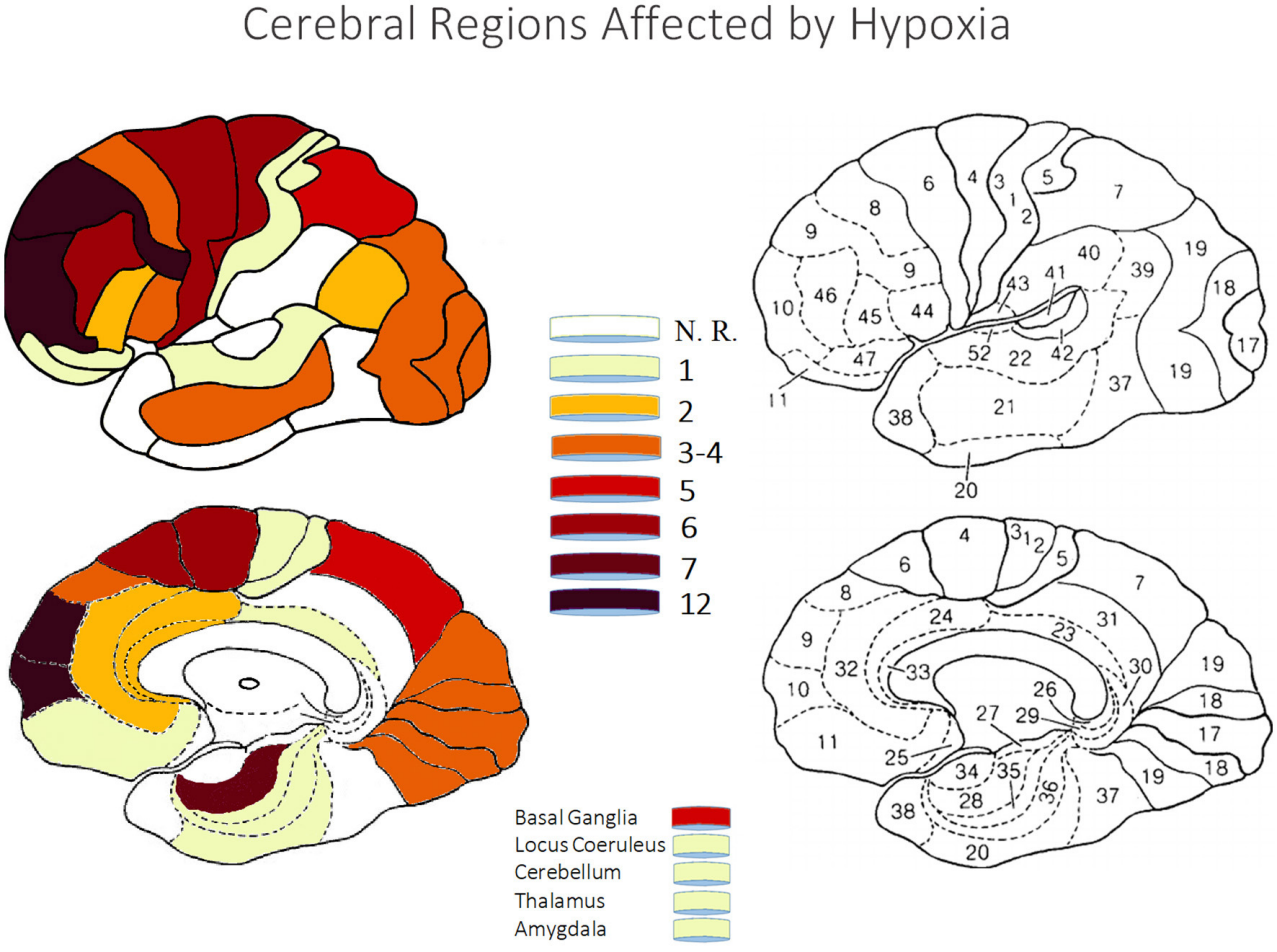
The figures show color-coded dorso-lateral and medial sagittal brain maps (**left**: top and bottom, respectively) indicating the frequency with which the manifold Brodmann areas (BA; **right**) were reported to be affected by the different types of normobaric or hypobaric hypoxia in the studies listed in Table 1. Affected areas include reduced metabolism, altered connectivity and increased activation, as well as brain damage (see also Tables 2, 3). Note that the mesencephalic areas indicated are not shown graphically, but are simply reported by color coding. N. R., no report.

To gain an overview of the main effects of HH or NH, regardless of the type of technique used and the experimental paradigm, we performed a selective search on Pubmed using brain and hypoxia as keywords (see Table 1 for a complete list of papers and for a description of their technical specifications). Criteria for inclusion were that the study was on humans (with the exception of a few rat brain dissection studies supporting the human findings), the altitude or hypoxia conditions were clearly specified, as were the experimental conditions of the task to be performed and the brain areas involved, with precise specification of the Brodmann areas (BA). Based on this information, we created colorimetric anatomical frequency maps to highlight the brain areas most susceptible to hypoxia on the two cerebral hemispheres, regardless of the hypoxic mode. These maps are visible in Figure 4.

**Table 1:** Review of papers showing functional and clinical effects of hypobaric or normobaric hypoxia.

**Authors**	**Technique**	**Paradigm**	**Brain areas**	**Functional effects**	**Hypoxia conditions**
Bhattacharjee et al. (2023)	Brain dissection in rats	Motor activity, spatial memory	Prefrontal cortex, Hippocampus, basal ganglia, locus coeruleus, cerebellum	Significant alteration of the catecholamine levels	Simulated altitude of 22,000 ft (6,706 m a.s.l.)
Brierley (1976)	Various	Review of studies	Hippocampus, thalamus, amygdala, striatum	Brain damage	Hypoxia
Buck et al. (1998)	PET	Resting	Increased blood flow in the hypothalamus	Change in metabolism	Acute normobaric hypoxia (3,000–4,500 m; 14,746 ft-20,998 ft a.s.l.)
Davranche et al. (2016)	fNIRS	Simon task (cognitive conflict)	Prefrontal cortex	Increased activation (workload)	Exposure at HA (4,350 m; 14,272 ft a.s.l.)
Di Paola et al. (2008)	MRI voxel-based morphometry	Resting	Left BA4, SMA BA6, Angular gyrus BA39	Reduced WM density/volume	Repeated and acute HA exposure
Fayed et al. (2006)^*^	Single-voxel MR spectroscopy	Resting	Bilateral frontal and basal ganglia	Brain damage	HA climbing without oxygen
Garrido et al. (1993)	MRI	Resting	Visual cortex	Cortical atrophy	Over 8,000 mt climbing without oxygen
Garrido et al. (1995)^**^	MRI	Resting (Several altinauts reported hallucinating while climbing)	Right occipital lobe	Lesion located in the white matter of the occipital lobe	Over 8,000 m climbing without oxygen
Hochachka et al. (1999)	PET	Resting	Frontal lobes	Reduced metabolism	Permanence at HA (3,700–4,900 m; 12,139–16,076 ft a.s.l.)
Hutcheon et al. (2023b)	EEG	Attentional orienting	Occipito/parietal	EEG Alpha decrease	Acute hypobaric hypoxia
Kauser et al. (2016)	Bright field microscopy	Resting	Medial Prefrontal cortex (mPFC)	Brain damage	HA hypobaric hypoxia (7620 m; 25,000 ft a.s.l.).
Kumari et al. (2018)	Brain dissection	Cued and contextual fear conditioning in rats	Medial PFC, hippocampus, limbic area	Brain damage	Simulated HA exposure (25,000 ft; 7620 m a.s.l.)
Lieberman et al. (1995)	Neurological assesment	Speech production	Fronto-striatal circuits BA4, BA9, BA7	Disruption of subcortical pathways to the prefrontal cortex	Exposure to HA (Mt. Everest) hypobaric hypoxia
Lieberman et al. (2005)	Neurological assesment	Speech production	Hippocampus, basal ganglia	Brain damage	Exposure to HA hypobaric hypoxia
Ma et al. (2021)	ERPs and sLORETA	Attentional orienting	Medial frontal cortex, precuneus	Reduction of the executive functions	Long-term exposure to HA
Manferdelli et al. (2021)	NIRS	Constant-speed exercise	DLPF cortex	Reduced prefrontal cerebral oxygenation	Normobaric hypoxia (3,647 m; 11,965 ft a.s.l.)
Minamoto et al. (2024)	fNIRS and GLM-based approach	Self referential task	Change in default-mode network (DMN) connectivity	Altered connectivity in cortical midline structures	Normobaric hypoxia
Ochi et al. (2018)	fNIRS	Stroop task (conflict)	Left DLPF cortex	Decreased activation	Acute normobaric hypoxia (3,500 m; 11,483 ft a.s.l.)
Pagani et al. (2000)	SPECT	Resting	Increased rCBF in sensorimotor and prefrontal cortices	Altered metabolism in temporal, parahippocampal, parietal and central areas structures	Acute hypobaric hypoxia
Pagani et al. (2011)	rCBF	Resting	Anterior areas BA4, BA6, BA 44/45	Reduced metabolism	Acute hypobaric hypoxia
Pichiule et al. (1996)	Brain dissection in rats	Coercion	Cerebral cortex, hippocampus and corpus striatum of 3 week old rats	Severe reduction in NMDA binding site	Intermittent hypobaric hypoxia (4,300 m; 14,108 ft a.s.l.)
Rogan et al. (1922)	1 h fMRS, rCBF study	Resting vs. delayed memory task	Global rCBF increase	rCBF decrease in posterior cingulate cortex (PCC) (BA23)	Two hours acute normobaric hypoxia (,5505 m; 18,130 ft a.s.l.)
Rozhkov et al. (2009)	EEG/dipole modeling	Resting	Medial temporal lobe, limbic system	Increase in dipole strenght	Acute hypoxia (92% nitrogen breathing)
Soroko et al. (2007)	EEG/dipole modeling	Resting	Frontal lobes, medial temporal, limbic system	Increased bilateral EEG asynchronism of Theta and Delta waves	Acute hypoxia (92% nitrogen breathing)
Subudhi et al. (2007)	NIRS	Incremental cycling exercise	BA6, BA44	Cerebral deoxygenation	Acute normobaric [12% Fi(O_2_) hypoxia]
Tuunanen et al. (2006)	fMRI	Checkerboard stimulation	Visual cortex (BA17/18)	BOLD signal reduction	Mild hypobaric hypoxia
Vestergaard et al. (2016)	fMRI	Resting	Visual cortex	Increased rCBF (hyperventilation)	Acute hypoxia
Virués-Ortega et al. (2004)	Various	Review of studies	Frontal lobe, middle temporal lobe	Brain Damage	Exposure to HA
Wang et al. (2021)	ERP	Visual oddball (letters)	BA10, BA11, BA47	Reduced volume and metabolism	Long term hypoxia
Wang et al. (2022)	fNIRS	Working memory	DLPF cortex	Abnormal hemodynamic responses (increase/decrease)	Acute HA exposure
Yan et al. (2011)	fMRI	2-back verbal working memory task	IFG, MFG, MOG, lingual gyrus	Decreased brain activation, longer RTs, lower accuracy in behavioral performance	Impact of HA residence since birth [i.e., 2,616–4,200 m (8,780–13,780 ft)]^***^
Yan et al. (2011)	fMRI	2-back spatial working memory task	STG BA 22, MOG BA19, pyramids of vermis	Decreased brain activation, longer RTs, comparable accuracy in behavioral performance	Impact of HA residence since birth [i.e., 2,616–4,200 m (8,780–13,780 ft)]^***^
Zani et al. (2019)	ERPs and sLORETA	Attentional workload	Left parahippocampal area BA35–36, ACC BA24	Larger ERPs, dipole strength increase	Acute normobaric hypoxia
Zani et al. (2023)	ERPs and sLORETA	Attentional orienting	Bilateral SFG BA10, left precuneus BA7, left MTG BA37/19, right ACC BA32	Larger ERP responses (effort)	Acute normobaric hypoxia

^*^MRI brain scans performed in a group of mountaineers after they returned from climbing expeditions found cortical atrophy and diffuse enlargement of Virchow-Robin spaces in the basal ganglia and semioval center. Multiple subcortical lesions were found in some mountaineers, which persisted in association 3 years later. Radiological evidence was also found of some irreversible lesions in the brains of amateur mountaineers and cortical atrophies in professionals, thus suggesting chronic damage.

^**^Several of the climbers tested reported hallucinations during the climbing.

^***^ With relocation at an altitude of <400 m for 1 year vs. a.s.l. permanent residents.

ACC, anterior cingulate cortex; a.s.l., above sea level; BA, Brodmann area; Behav Perf, behavioral performance; BOLD, blood oxygen level dependent; DMN, default mode network; DLPF, Dorso lateral prefrontal; EEG, electroencephalogram; Electroencephalography; EEG/dipole modeling, a search for intracerebral sources of bioelectrical potentials measured at the scalp; ERP, event related potentials; fMRI, functional magnetic resonance imaging; fMRS, functional magnetic resonance spectroscopy; fNIRS, functional near infrared resonance spectroscopy; GLM-based approach, general linear model based approach; IFG, inferior frontal gyrus; HA, high altitude; mPFC, medial prefrontal cortex; MFG, Middle Frontal Gyrus; MOG, middle occipital gyrus; MRI, magnetic resonance imaging; MTG, middle temporal gyrus; NMDA, N-methyl-D-aspartate receptor; rCBF, regional cerebral blood flow; PET, positron emission tomography; SPECT, single photon emission computerized tomography; SPL, superior parietal Lobule; WM, white matter.

Overall, the techniques used in the studies were diverse and encompassed various bioelectric and hemodynamic imaging as well as neurological assessment methods. Positron Emission Tomography (PET) and functional Near-Infrared Spectroscopy (fNIRS) were commonly utilized, alongside Magnetic Resonance Imaging (MRI), which included voxel-based morphometry and single-voxel MR spectroscopy. Electroencephalography (EEG), often coupled with dipole modeling, was another frequently used technique. Some studies incorporated bright field microscopy and neurological assessments. Event-Related Potentials (ERPs) and standardized Low-Resolution Electromagnetic Tomography Analysis (sLORETA) were used to analyze brain activity, while Single-Photon Emission Computed Tomography (SPECT) and regional Cerebral Blood Flow (rCBF) studies provided insights into brain perfusion. Functional MRI (fMRI) was also extensively used, with some studies employing a GLM-Based Approach (see acronym in Table 1 notes) to analyze fNIRS data. Additionally, a 1-h functional Magnetic Resonance Spectroscopy (fMRS) combined with rCBF study was conducted to examine brain function in greater detail. Researchers applied various other techniques across different studies to ensure a comprehensive understanding of the neurological and cognitive processes under investigation (e.g., brain dissection on rats).

Since EEG/ERPs and ERF have an advantage in temporal resolution over haemodynamic neuroimaging techniques that, conversely, have an advantage in spatial resolution over the former research tools (Zani et al., 2003; Purves et al., 2008), the study of brain dynamics at the level of the intracranial source can allow a valuable comparison of findings between techniques and a faster detection of changes due to hypoxia or other CNS activations and/or deactivations reported by haemodynamic studies. In principle, the study of intracranial sources, or the combination of this study with other imaging techniques, can provide a more accurate and reliable view of the distributed changes in activity of brain centers and networks during hypoxic exposure. Possibly, these changes would not merely be related to the specific areas that characterize the widely reiterated paradigms in the literature (e.g. oddball, preattentive and/or attentive paradigms, working memory, etc.).

In this regard, no matter the bioelectrical and/or haemodynamic imaging mode, researchers suggest that the most damaged brain structures include the hippocampus, thalamus, and layers 2, 4, and 5 of the cortex (see Table 1). The high sensitivity of structures like the hippocampus and limbic system to hypoxia results in changes to cognitive functions associated with these areas. Lesions in the medial temporal lobe, hippocampus, and para-hippocampal regions can lead to disorders in the declarative memory system, affecting the ability to consciously retrieve and access memories (Zola-Morgan et al., 1986; Fell et al., 2001).

A partial infarction of the periventricular white matter results from leukoaraiosis, the rarefaction of the brain white matter caused by loss of axons and myelin due to ischemic injury and appears at the tissue level, which also causes demyelination, axonal degeneration, and astrogliosis (Virués-Ortega et al., 2004). For those subjected to extended or frequent exposure to high altitudes, extreme desaturation therefore results in neuronal death. Hemodynamic neuroimaging findings associate reduced activity in the anterior cingulate cortex (ACC) with activation of the prefrontal cortex in children with behavioral control disorder (Goldberg et al., 2011).

Immigrants from high altitudes also show a significant decrease in gray matter volumes in the inferior and middle frontal gyrus and in the ACC, as well as a decrease in the left middle occipital gyrus. ERPs, which have a temporal advantage over neuroimaging techniques, also report cortical changes, showing reduced N2 (a neural marker of attention and behavioral inhibition) and P3b amplitudes but increased ERN and CRN amplitudes (Ma et al., 2015) in individuals residing at high altitudes (Wang et al., 2021). In terms of processing conflicting information, researchers have found a smaller N2 difference wave between congruent and incongruent target stimuli for people living at the highest altitude of the three HA levels studied, namely 2,700, 3,700 and 4,500 m (Ma et al., 2019). In addition to evidence from EEG and ERPs, magnetic resonance spectroscopy (MRS) and other hemodynamic indices have been used to study the risks of Hypoxia and brain injury in altinauts.

In general, our analysis indicated that effort associated with task demands in hypoxic conditions revealed increased activation of brain areas (e.g., of prefrontal cortex). The brain areas that showed this increased activation or metabolism are listed in Table 2. However, in most cases, the functional effects were a reduced activation of a specific area, reduced metabolism or hemodynamic response, an impairment of a specific function, or a functional lesion as a result of hypoxia (see Table 3).

**Table 2:** Brain areas most likely to show increased brain activation or metabolism due to hypoxia, based on studies reviewed in our analytical assessment of the literature (Table 1).


1. Prefrontal cortex—Increased activation (workload) 2. Hypothalamus—Increased blood flow and change in metabolism 3. Sensorimotor and prefrontal cortices—Increased regional cerebral blood flow (rCBF) 4. Medial temporal lobe and limbic system—Increase in electric dipole strength 5. Visual cortex (BA17/18)—Increased rCBF (hyperventilation) 6. Posterior Cingulate Cortex (PCC) (BA23)—Global rCBF increase (with a decrease in PCC) 7. Left Parahippocampal area (BA35-36), anterior cingulate cortex (ACC) (BA24)—Larger Event-related potentials (ERPs), increased dipole strength 8. Bilateral superior frontal gyrus (SFG) (BA10), left precuneus (BA7), left middle temporal gyrus (MTG) (BA37/19), right ACC (BA32)—Larger ERP responses (effort)

**Table 3:** Brain areas mostly showing decreased or impaired brain activation or hypo-metabolism due to hypoxia.

**1. Prefrontal cortex—Reduced metabolism, decreased activation, abnormal hemodynamic responses (increase/decrease), impaired activation in BA10, BA11, BA47**
2. Hippocampus—Brain damage, decreased activation along with the limbic area
3. Basal ganglia—Brain damage, decreased activation along with hippocampus
4. Frontal lobes—Reduced metabolism, brain damage, increased bilateral EEG asynchronism of Theta and Delta waves
5. Occipital lobe—Lesion in right occipital white matter, cortical atrophy in the visual cortex, BOLD signal reduction in visual cortex (BA17/18)
6. Medial prefrontal cortex (mPFC)—Brain damage
7. Dorsolateral prefrontal (DLPF) cortex—Reduced prefrontal cerebral oxygenation, decreased activation, abnormal hemodynamic responses
8. Fronto-striatal circuits (BA4, BA9, BA7)—Disruption of subcortical pathways to the prefrontal cortex
9. Medial PFC, hippocampus, limbic area—Brain damage
10. Medial Frontal cortex, precuneus—Reduction of executive functions
11. Anterior areas (BA4, BA6, BA44/45)—Reduced metabolism, cerebral deoxygenation
12. Cerebral cortex, hippocampus, corpus striatum in rats—Severe reduction in NMDA binding sites.
13. Middle temporal lobe—Brain damage, decreased activation in left MTG (BA37/19).
14. Inferior Frontal Gyrus (IFG), Middle Frontal Gyrus (MFG), Middle Occipital Gyrus (MOG), Lingual gyrus—Decreased brain activation, longer reaction times (RTs), lower accuracy in behavioral performance
15. Superior Temporal Gyrus (STG) (BA22), MOG (BA19), Decreased brain activation, longer RTs, comparable accuracy in behavioral performance
16. Occipito/parietal regions—EEG Alpha decrease

Researchers varied the conditions of hypoxia among different forms and altitudes, including simulated altitude of 22,000 ft (6,706 m a.s.l.), acute NH typically between 3,000–4,500 m (14,746–20,998 ft a.s.l.), and exposure at 4,350 m (14,272 ft a.s.l.). There were instances of repeated and acute HA exposure, such as climbing without oxygen above ~8,000 m (~26,247 ft) m and extended stays at high altitudes between 3,700–4,900 m (12,139–16,076 ft a.s.l.). Acute hypobaric hypoxia conditions were experienced at 7,620 m (25,000 ft a.s.l.), similar to those found on Mt. Everest, and long-term HA exposure was also considered. Additionally, NH was observed at 3,647 m (11,965 ft a.s.l.), with specific cases of acute hypoxia involving 92% nitrogen breathing or 12% Fi(O_2_) levels. The impact of residing at high altitudes since birth (2,616–4,200 m) and relocating to lower altitudes (<400 m) was compared to that of permanent sea-level residents. The effects of intermittent HH at 4,300 m (14,108 ft a.s.l.) and short-term (2-h) acute NH at 5,505 m (18,130 ft a.s.l.) were also examined. These conditions covered a range of hypoxia and HA exposures, both short and long term, with variations in atmospheric conditions and oxygen levels.

The experimental paradigms ranged from various cognitive and physical activities to resting states. Motor activity and spatial memory were assessed, as well as attentional orienting and working memory through tasks such as the Simon task (cognitive conflict), Stroop task (conflict), and visual oddball (letters). Speech production was also examined. Physical activities included constant-speed exercise and incremental cycling exercise. Self-referential tasks and coercion were part of the cognitive evaluations, and cued and contextual fear conditioning were tested in rats. Resting states were frequently studied, sometimes alongside tasks like delayed memory tasks. In some resting conditions, participants experienced hallucinations during climbing. The paradigms included working memory tasks like the 2-back verbal and 2-back spatial working memory tasks, as well as attentional workload assessments. Finally, checkerboard stimulation was also part of the experimental conditions.

The brain areas that showed impaired activation, decreased activation, or functional lesions are listed in Table 3.

The five areas most affected by hypoxia (as also reported in Table 4), were: (i) the prefrontal cortex and frontal lobe, responsible for complex cognitive behavior, such as decision-making, goal-directed movement, social behavior, personality expression, and moderating social behavior. The prefrontal cortex plays a crucial role in executive functions such as planning, reasoning, and problem solving (Stuss and Knight, 2013; Miller and Cohen, 2001). (ii) Hippocampus, critical for the formation of new memories and associated with learning and spatial memory (Eichenbaum, 2017). (iii) Basal Ganglia, involved in the regulation of voluntary motor movements, procedural learning, routine behaviors or “habits,” movements coordination (Graybiel, 2008). (iv) Frontal lobes related to reduced metabolism, altered hemodynamic responses and increased bilateral EEG asynchronisms of Theta and Delta oscillations. (v) Occipital Lobe/Visual Cortex, primarily responsible for visual processing, enabling us to interpret shapes, colors, and motion (Wandell and Smirnakis, 2009).

**Table 4:** Opposite or different effects of NH vs. HH on brain functions.

**1. Prefrontal cortex •Normobaric hypoxia: increased activation (workload), reduced metabolism, abnormal hemodynamic responses •Hypobaric hypoxia: reduction of executive functions and altered connectivity in cortical midline structures**
2. Hippocampus •Normobaric hypoxia: brain damage, reduction in NMDA binding sites •Hypobaric hypoxia: brain damage, decrease in activation along with basal ganglia
3. Basal ganglia •Normobaric hypoxia: brain damage, disruption of subcortical pathways to the prefrontal cortex •Hypobaric hypoxia: brain damage, disruption of subcortical pathways to the prefrontal cortex
4. Frontal lobes •Normobaric hypoxia: reduced metabolism, abnormal hemodynamic responses •Hypobaric hypoxia: brain damage and increased bilateral EEG asynchronism of Theta and Delta waves
5. Visual cortex •Normobaric hypoxia: BOLD signal reduction, increased rCBF with hyperventilation •Hypobaric hypoxia: Cortical atrophy, BOLD signal reduction

These areas were significantly impacted by hypoxia, leading to various degrees of functional impairment, including visual hallucinations (Harris et al., 2018), poor decision making (Wilson et al., 2009), impaired or altered cognitive functions encompassing attention, vigilance, perception, judgment and working memory. From the data, it appears that both normobaric and hypobaric hypoxia conditions affected similar brain areas, albeit with some variations (see Table 4 again).

## Discussion and conclusions

Hypoxia impacts psychological states and cognitive functions. According to Brownlee et al. (2020), the domains most affected are executive functions and short-term memory recall, including declines in visuo- and audio-spatial skills, processing speed, planning, and attention. Hypoxia also disrupts motor coordination, demonstrated by the Finger Tapping Test. Severe hypoxia is linked to functional impairments in regions like the frontal lobe and temporal lobe, showing effects on language and comprehension, especially above 6,400 m (~20,997 ft).

The review highlights significant differences between NH and HH. NH primarily affects brain activation patterns, while HH shows structural damage in areas such as the hippocampus and basal ganglia. Both forms impair cognitive functions, but HH tends to produce more severe effects.

Indeed, in terms of the main deficits observed, exposure to hypoxia, in whatever form, such as mountaineering, decompression of airplane cockpits, diving, respiratory insufficiency, etc., can lead to cognitive impairments such as reduced attention, impaired judgement and compromised judgement and decision-making abilities.

These deficits worsen with limited oxygen availability, affecting high cognitive centers like the prefrontal cortex (e.g., Wilson et al., 2009). Additionally, HA mountaineering is linked to visual hallucinations due to changes in visual processing regions, such as the primary occipital cortex (Harris et al., 2018). Visual processing and perception are significantly impacted by hypoxia, especially in high-altitude environments (e.g., Altbäcker et al., 2019; Blacker et al., 2021).

These deficits increase with limited oxygen availability, particularly impacting high cognitive centers like the prefrontal cortex. Visual hallucinations due to changes in processing regions like the primary occipital cortex are noted, while auditory processing deficits receive less emphasis despite being documented (e.g., Lucertini et al., 2020, 2002).

EEG markers of hypoxia effects such as alpha and gamma rhythms reflect attentional orientation and working memory deficits. Hypoxia also affects neural processing involved in orienting attention and analyzing target stimuli, altering ERP components including ADAN, LDAP, P3a, MMN and P3b.

Ascending to high altitudes poses risks like brain lesions, atrophy, and neurocognitive alterations, especially for poorly acclimatized individuals. Acute Mountain Sickness (AMS) and High-Altitude Cerebral Edema (HACE) are significant challenges above 2,500 m (~8,202 ft), with AMS characterized by symptoms like headache and dizziness (Lawley et al., 2014). Research indicates that high-altitude exposure impairs memory and slows reaction times, with effects varying by altitude and duration. Notably, acclimatization can moderate these cognitive impacts, as shown by improved memory performance in mountaineers at 5,350 m (Pagani et al., 1998).

## Future directions

Future research should address auditory and other sensory deficits alongside visual alterations to provide a more complete understanding of hypoxia's sensory impact. Including a diverse range of participant populations beyond specific cohorts, such as mountaineers, will enhance the generalizability of findings to various environments and individuals with different pre-existing conditions.

Additionally, investigations should focus on identifying effective cognitive recovery strategies following hypoxic exposure, as cognitive impairment can significantly vary based on acclimatization, physical condition, and duration of exposure. Lastly, exploring the long-term consequences of hypoxia-induced brain changes is essential for understanding chronic implications on cognitive health and developing preventive or rehabilitative measures.

## Footnotes

^1^AMS must not be confused with high-altitude cerebral edema (HACE). AMS alone exhibits no neurological findings and is self-limited. In contrast, HACE, which usually comes on between 24 and 72 h after a gain in altitude, is characterized by change in mental status and/or ataxia, occurs usually in a person with AMS or high-altitude pulmonary edema, and is a medical emergency (Hackett and Roach, 2004; Willmann et al., 2014).
